# Field Monitoring of Colostral BVDV-, BoHV-1-, and BRSV-Specific Serum Antibody Levels in Dairy Calves from Birth to Weaning Fed with Pasteurized Colostrum Pools Obtained from Vaccinated Dams

**DOI:** 10.3390/vaccines13070709

**Published:** 2025-06-29

**Authors:** Veysel Soydal Ataseven, Ufuk Kaya, Müge Doğan, Sultan Şengül, Seda Turan, Fatma Türkarslan Akbaba, İsmail İlker Kocaer

**Affiliations:** 1 Department of Virology, Faculty of Veterinary Medicine, Hatay Mustafa Kemal University, 31060 Hatay, Türkiye; 2 Department of Biostatistics, Faculty of Veterinary Medicine, Hatay Mustafa Kemal University, 31060 Hatay, Türkiye; u.kaya@mku.edu.tr; 3 Laboratory of Virology, Konya Veterinary Control Institute, 42090 Konya, Türkiye; muge.dogan@tarimorman.gov.tr; 4 AtaSancak Acıpayam Agricultural Enterprise, 20800 Denizli, Türkiye; vetsultan49@gmail.com (S.Ş.); sedaturan.0502@gmail.com (S.T.); ilkerkocaer@atasancak.com.tr (İ.İ.K.); 5 Laboratory of Bacteriology, Konya Veterinary Control Institute, 42090 Konya, Türkiye; fatmaturkarslan@gmail.com

**Keywords:** bovine herpesvirus-1, bovine viral diarrhea virus, bovine respiratory syncytial virus, colostrum, passive immunity, socialization, stress, vaccination

## Abstract

**Background/Objectives**: This study aimed to determine the changes in BVDV (bovine viral diarrhea virus), BoHV-1 (bovine herpesvirus-1), and BRSV (bovine respiratory syncytial virus) antibody levels until weaning in calves who ingested colostrum from vaccinated dairy cattle. Additionally, it aimed to measure the antibody levels induced by the vaccine administered before and after socialization after weaning. **Methods**: Exposure to respiratory viral and bacterial agents was monitored by PCR analysis using nasal swabs at regular intervals from birth to weaning (pre-colostral and after the 2nd, 7th, 15th, 25th, 35th, 45th, 55th, and 65th days). The levels of colostral BVDV, BoHV-1, and BRSV antibodies were monitored using an enzyme-linked immunosorbent assay (ELISA) at the same intervals from birth to weaning (pre-colostral and after the 2nd, 7th, 15th, 25th, 35th, 45th, 55th, and 65th days). **Results**: The highest level of maternal antibodies in the blood was detected on day 7. BoHV-1, BVDV, and BRSV antibody levels decreased steadily until weaning by 69.14%, 38%, and 53%, respectively. **Conclusions**: Vaccination strategies should be planned by considering the presence of maternally derived antibodies and minimizing stress that may negatively affect vaccine titers, thus maximizing vaccine efficacy in calves.

## 1. Introduction

In some cases, viral and bacterial agents interact with each other in ways that can disrupt homeostasis, such as stress. Together, they form the bovine respiratory disease (BRD) complex [1,2]. Examples of these agents include bovine herpesvirus-1 (BoHV-1), bovine viral diarrhea virus (BVDV), bovine parainfluenza virus-3 (BPIV-3), bovine respiratory syncytial virus (BRSV), bovine coronavirus (BCoV), *Mannheimia haemolytica* (*M. haemolytica*), *Pasteurella multocida* (*P. multocida*), *Histophilus somni* (*H. somni*), *Mycoplasma bovis* (*M. bovis*), and *Trueperella pyogenes* (*T. pyogenes*). Although these viral pathogens can cause respiratory disease of varying clinical severities alone, they can predispose the lungs to secondary bacterial infections if viral replication is not controlled [3]. For example, BoHV-1 and BVDV infections play an important role in reproductive problems such as infertility, abortion, repeat breeding, and neonatal calf mortality, in addition to respiratory system infections [3,4,5]. BRSV, a pneumovirus that typically causes acute respiratory system infection, does not have a persistence feature like BVDV and BoHV-1, although all three viruses can mediate immunosuppressive effects through different mechanisms [6,7].

Although BRDs can affect cattle of all ages and at different production stages, they are frequently seen in the neonatal period because calves are born agammaglobulinemic; they are also seen close to weaning age, when immunity is suppressed due to intense stress [8,9]. By affecting live weight gain and fertility, respiratory infections in calves from birth to weaning age have a critical impact on future productivity. The main factor that protects calves during this period includes immunoglobulins that are passively transferred from dams via colostrum. These immunoglobulins, in addition to providing systemic immunity and non-nutritional factors like leukocytes, hormones and growth factors, oligosaccharides, and microRNAs in colostrum, also significantly influence the maturation of the calf’s intestinal and systemic immune functions [10]. However, colostrum quality, which is the primary criterion for successful passive transfer, is assessed by immunoglobulin concentration and is affected by factors such as the degree to which cattle within the herd are exposed to field strains, herd vaccination programs, the frequency of vaccination, responses to vaccination, and the ability to transfer specific antibodies to colostrum [11,12,13,14]. Vaccinations in the last trimester of pregnancy increase the immunoglobulin (Ig) levels in colostrum and calf blood serum. Thus, the increase in colostrum antibody levels is largely related to the increase in antibody levels in calf blood serum [15]. In addition, the timing of colostrum intake and the method and volume of colostrum administration also affect the optimization of absorption [16].

While maternal antibodies in calves can be detected at 1–6 months [12,17], antibody titers as a parameter may not accurately reflect disease protection [18]. Therefore, in the present study, we observed colostral antibody dynamics in a commercial dairy cattle enterprise under field conditions rather than determining the protective efficacy of maternal antibodies. The aim of this study was to determine changes in BVDV, BoHV-1, and BRSV serum antibody levels in colostrum from the neonatal period to weaning in calves. These calves were born to dairy cattle regularly vaccinated with a commercial vaccine containing inactivated BVDV, BoHV-1, and BPIV-3 and live attenuated BRSV at 6-month intervals. Although colostrum provides protection against infections in the first period of the calf’s life, the continuity of this protection can only be achieved through vaccination. Accordingly, the second phase of the study measured antibody levels induced by the vaccine administered before and after socialization following weaning.

## 2. Methods

### 2.1. Monitoring Study

A total of 32 calves in one dairy cattle enterprise were monitored for the dynamics of three infections and the levels of colostral BVDV, BoHV-1, and BRSV antibodies at regular intervals from birth until weaning (days 2, 7, 15, 25, 35, 45, 55, and 65). Exposure to some respiratory viral and bacterial agents (BVDV, BRSV, BPIV-3, BoHV-1, BCoV, *M. haemolytica*, *P. multocida*, *M. bovis*, and *T. pyogenes*) during the study period was also monitored by a PCR analysis of nasal swabs.

Whole blood and serum samples collected from pre-colostral calves were examined for the presence of BVDV antigens and BoHV-1-, BVDV-, and BRSV-specific antibodies before feeding with a pasteurized colostrum pool at 25% brix obtained from multiparous cattle regularly vaccinated at 6-month intervals with a commercial vaccine (Hiprabovis 4; HIPRA Laboratorios, Amer, Spain). Each calf was given 4 L of pasteurized colostrum within 15 min after birth and then fed with 2 L of the same colostrum every 4 h. The calves were fed with formula food from the second day onwards.

An authorized veterinarian reported that an attenuated live intranasal BRSV vaccine (NASYM, HIPRA Laboratorios, Amer, Spain) and an inactivated vaccine containing *H. somni* and *M. haemolytica* leukotoxoid (Hiprabovis Somni/Lkt, HIPRA Laboratorios, Amer, Spain) were administered to the calves on days 2 and 33, respectively.

### 2.2. Socialization Groups and Vaccination Design

In the second phase of the study, the animals were divided into two groups. The 16 calves in group I were moved from their individual paddocks to the same area as 194 calves vaccinated with the first dose before socialization (total group size: 210 calves), and the calves in group 1 were vaccinated 1 week later. The 16 group II animals were moved from their individual paddocks 1 week after receiving the vaccine to the same area as the 194 calves vaccinated with the first dose before socialization (total group size: 210 calves). Serum and nasal swab samples were taken from calves in both groups on days 0, 7, 15, and 21 of the vaccination study. All calves were vaccinated with the same commercial vaccine (Hiprabovis 4; HIPRA Laboratorios, Amer, Spain).

### 2.3. Analysis of Serum and Colostrum Antibody Levels and BVDV Antigen

Detection of anti-BVDV antibodies (BVDV Total Antibody ELISA; IDEXX, Liebefeld-Bern, Switzerland) in 1:100 dilutions and anti-BoHV-1 glycoprotein B (gB) antibodies (ID Screen IBR gB competition ELISA, ID.Vet, Grabels, France) in 1:2 dilutions was performed on the sera and colostrum packages using commercially available registered kits in Türkiye. Anti-BRSV-specific antibodies in serum and colostrum samples were analyzed using a commercial ELISA kit (Monoscreen Antibody BRSV ELISA, Bio-X Diagnostics, Rochefort, Belgium) in 1:100 and 1:4 dilutions, respectively. The cut-off values of the ELISA kits for detecting BoHV-1-, BVDV-, and BRSV-specific antibodies were defined as sample/positive (S/P) ratios of ≤45%, ≥0.35, and value (V) > 20%, respectively. Testing for BVDV antigens in whole blood samples was carried out using commercial ELISA kits (BVDV Ag/Serum Plus Capture ELISA; IDEXX, Liebefeld-Bern, Switzerland). All absorbance values were measured at 450 nm using the ELISA reader (Mindray MR-96A; Shenzhen Mindray Bio-Medical Electronics Co., Ltd., Shenzhen, China), as described by the manufacturers.

### 2.4. Isolation of M. haemolytica, P. multocida, T. pyogenes, and M. bovis

The nasal swab samples were cultured in enriched blood agar by adding 7% sheep blood. The media were then incubated at 37 °C in an aerobic environment for 24–48 h. The colony morphology, hemolysis properties, Gram staining, oxidase, catalase, and Mac Conkey agar growth characteristics of the bacteria grown in blood agar were identified according to standard methods [19]. Colonies suspected of being biochemically or phenotypically positive for *P. multocida* and *M. haemolytica* were transferred to brain heart infusion broth for molecular characterization and incubated at 37 °C for 24 h. To isolate *M. bovis*, the nasal swabs were cultured in modified Hayflick medium containing 10% horse serum [20].

### 2.5. Polymerase Chain Reaction (PCR) for Viruses and Bacteria

Viral nucleic acids were isolated from 200 µL of the calves’ nasal swab samples using a commercial kit (IndiMag Pathogen Kit, Indical Bioscience, Leipzig, Germany) in an automated extraction device (IndiMag 48s, Indical Bioscience, Leipzig, Germany) according to the manufacturer’s instructions. Extraction of the bacterial nucleic acids from the nasal swab samples and culture isolates was performed using a commercial kit (IndiSpin Pathogen Kit, Indical Bioscience, Leipzig, Germany) according to the manufacturer’s procedure in an automatic extraction device (QIAcube, Qiagen, Hilden, Germany).

Amplification procedures were performed using specific primers and probes, as described elsewhere (Table 1 and Table 2). Amplification was performed using commercial one-step qRT-PCR kits for BVDV, BRSV (IndiMix JOE kit, Indical Bioscience, Leipzig, Germany), BoHV-1, *M. haemolytica*, and *P. multocida* (The LightCycler^®^ 480 Probes Master kit, Roche Applied Science, Mannheim, Germany) according to each manufacturer’s recommendations.

All nasal swabs and isolate samples were tested for *T. pyogenes* and *M. bovis* DNA by conventional PCR using the primer sets described by Silva et al. [27] and Foddai et al. [28], respectively. For this, 6 μL of amplification products were run on a 1.5% agarose gel stained with GelRed (Biotium, Hayward, CA, USA) together with a 100 bp DNA marker (Solis Biodyne, Tartu, Estonia). The gel was then visualized using an Azure 280 Gel Imaging System (Azure biosystems, Dublin, OH, USA). The product sizes are shown in Table 2. Positive controls obtained from our laboratory were used in all the PCR analyses. Nuclease-free water was used as the negative control.

### 2.6. Statistical Analysis

Statistical analyses were performed using Stata 12/MP4 statistical software. The antibody levels of BVDV, BoHV-1, and BRSV were compared using parametric tests after confirming the data met parametric assumptions using the Kolmogorov–Smirnov test and Levene test for the normality and homogeneity of variances, respectively. The descriptive statistics were calculated as the arithmetic mean ± standard error of the mean. In the monitoring study, the differences between sampling times for each antibody level were determined using a mixed model for repeated measures design. The sampling times were included as the fixed factor, while the calves were included as the random factor in the model. For post hoc analysis, multiple comparisons were made using Bonferroni adjustment. The Pearson correlation coefficient was used to determine the relationship between antibody levels and those in colostrum at day 7. In the second phase of the study, the differences between sampling times for each antibody level were also determined, separately for groups I and II, in a mixed model for repeated measures design. The Mann–Whitney U test was used to compare antibody titers for BVDV, BoHV-1, and BRSV in groups I and II. In all statistical analyses, differences with *p* < 0.05 were considered statistically significant.

## 3. Results

### 3.1. Monitoring of Passive Immunity in Calves

None of the pre-colostral serum or whole blood samples obtained from the calves were positive for specific antibodies to the three viruses or the BVDV antigen, respectively. The interpretation values for anti-BRSV, anti-BVDV, and anti-BoHV-1 antibodies in colostrum were 125.1 of V, 2.1 of S/P, and 5.4 of S/P, respectively. As expected, there was a significant increase in specific antibody rates against the three viruses in all serum samples collected on day 2 after colostrum intake. The seroconversion determined on day 2 indicates that a completely healthy passive immune transfer occurred. Table 3 presents the relationships between each viral antibody level and colostrum antibody level on day 7.

BVDV antibody levels were highest on days 7 and 15 and significantly lower on day 2 (*p* < 0.05). Levels did not differ significantly between days 7, 15, and 25 (*p* > 0.05), but there was a significant decrease of 20.67% between days 25 and 35 (*p* < 0.05). Similarly, levels did not differ significantly between days 35 and 45 (*p* > 0.05), but there was a significant decrease between days 45 and 55 (*p* < 0.05). Overall, BVDV levels fell 38.39% from day 7 to day 65.

BoHV-1 antibody levels did not differ significantly between days 7, 15, and 25 (*p* > 0.05). However, levels were significantly lower on other days (*p* < 0.05) compared to day 7 (*p* < 0.05). More specifically, levels fell by 65.11% and 69.14% to a low value from day 7 to days 55 and 65, respectively.

**Figure 1:**
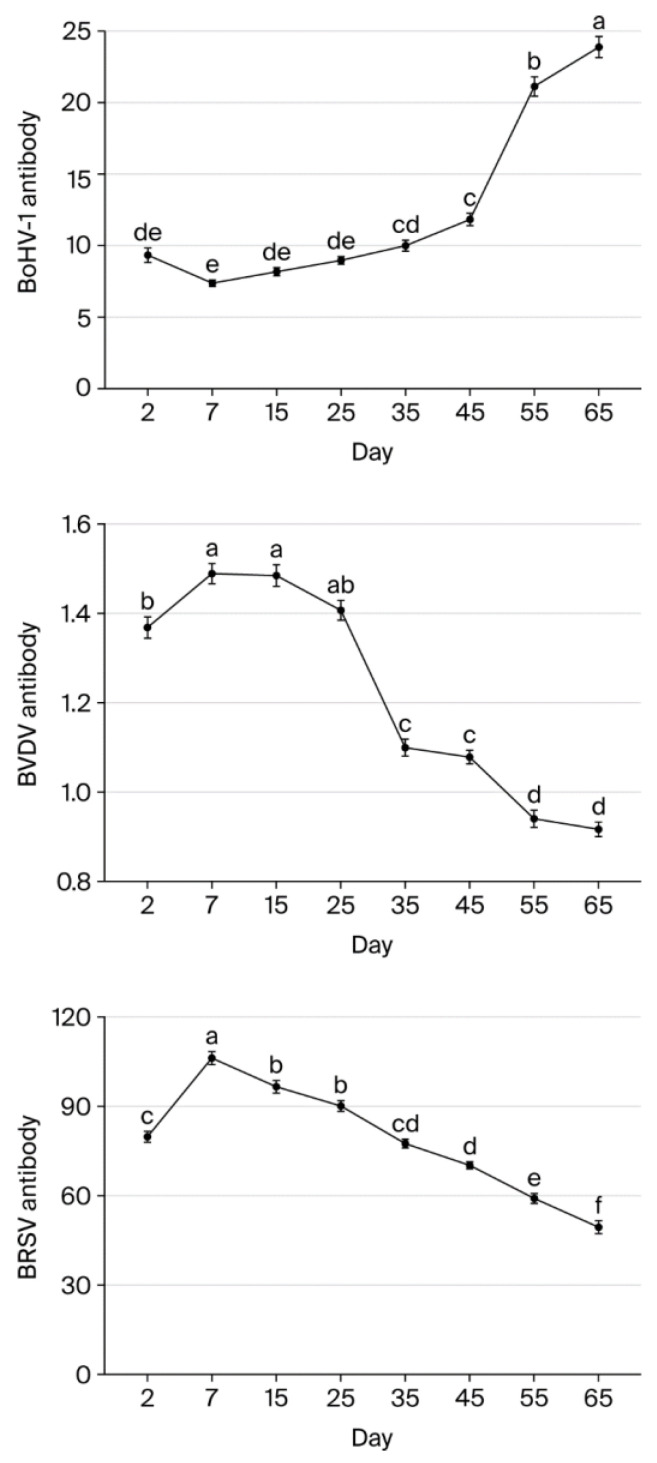
BoHV-1, BVDV, and BRSV antibody levels in calves on each sampling day (a, b, c, d, e, f—Different lowercase superscripts indicate statistically significant difference between sampling days).

BRSV antibody levels increased significantly by 24.86% between day 2 and day 7 (*p* < 0.05). After day 7, levels then declined gradually by 9.09%, 15.15%, 27.07%, 33.80%, 44.23%, and 53.37% on days 15, 25, 35, 45, 55, and 65, respectively. Overall, BRSV levels fell significantly from day 7 to day 65 (*p* < 0.05) (Table 4). Figure 1 and Table 5 present the changes in BVDV, BoHV-1, and BRSV antibody levels on each sampling day.

Of the 256 nasal swab samples analyzed with RT-PCR, all tested negative for BVDV, BoHV-1, and BRSV. However, BCoV nucleic acid was detected in one nasal swab from an apparently healthy calf on day 45. In addition, nasal swab samples from eight calves with respiratory distress, ranging from mild to severe pneumonia, on days 35, 45, 55, and 65 tested positive for *P. multocida* in six calves, *M. haemolytica* in one calf, and BCoV in one calf. None of the other bacterial or viral agents (*T. pyogenes*, *M. bovis*, BVDV, BRSV, BPIV-3, and BoHV-1) were detected in any swab samples (Table 6).

### 3.2. Monitoring of Humoral Immunity After Socialization and Vaccination

BVDV antibody levels in group I were highest on day 15 and significantly lower on days 0 and 7 (*p* < 0.05). On day 21, levels were similar to day 0 (*p* > 0.05), and they were at the lowest level on day 7 (*p* < 0.05). In group II, the levels were highest on day 15 and significantly lower on day 0 (*p* < 0.05). The levels were not significantly different on day 7, 15, or 21 (*p* > 0.05). BVDV antibody levels differed significantly between groups I and II on days 15 and 21 (*p* < 0.001 and *p* < 0.001, respectively).

In group I, pre-vaccination BVDV antibody levels increased in only two animals (2/16). In both of these animals, the levels decreased by day 15 before increasing in only one by day 21. In contrast, for the other animals in group I (14/16), the levels decreased before vaccination before increasing by day 15 and decreasing again after day 21 (15/16). In group II, BVDV antibody levels showed increases on days 7 and 15 in all animals (16/16) following vaccination before decreasing by day 21.

BoHV-1 antibody levels in group I were the highest on day 15 and significantly lower on days 0, 7, and 21 compared to day 15 (*p* < 0.05). The levels did not differ significantly on day 0 or 21 (*p* > 0.05) but were significantly lower on day 7 (*p* < 0.05). In group II, BoHV-1 antibody levels were highest on day 21 and did not differ significantly on day 7 or 15 (*p* > 0.05). On day 0, levels were significantly lower than on the other sampling days (*p* < 0.05).

BoHV-1 antibody levels did not differ significantly between groups I and II on day 15 (*p* > 0.05), but they did on day 21 (*p* < 0.001), with group II having a significantly higher level than group I. BoHV-1 antibody levels increased in all animals in both groups by day 15 after the first vaccination. However, while the levels showed decreases in all animals (16/16) in group I on day 21, they only showed a decrease in one animal (1/16) in group II.

**Figure 2:**
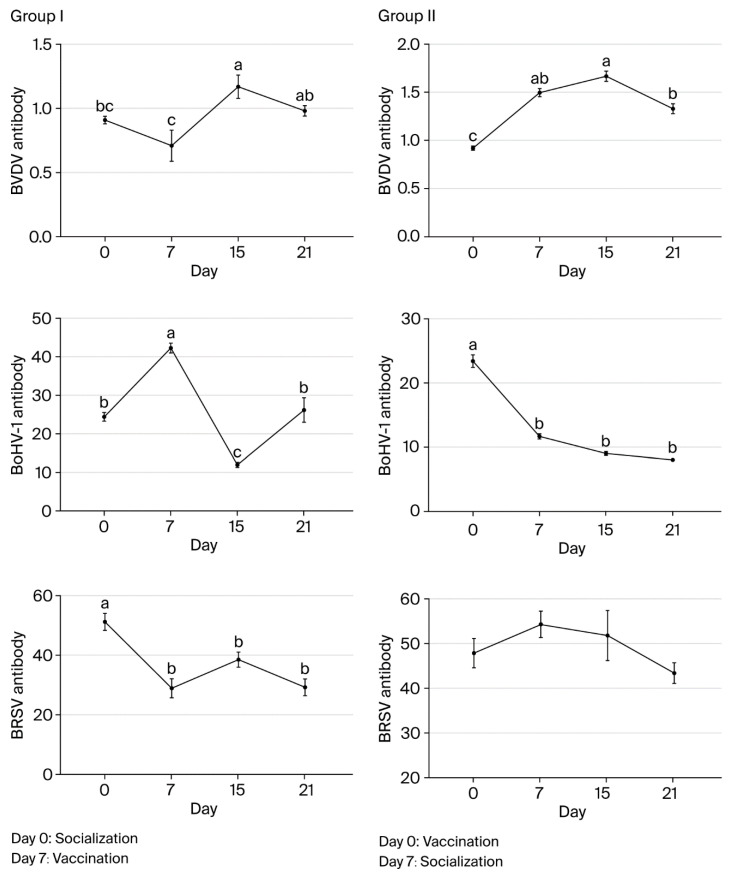
Changes in BVDV, BoHV-1, and BRSV antibody levels associated with socialization and vaccination (a, b, c—Different lowercase superscripts indicate statistically significant difference between sampling days).

BRSV antibody levels did not differ significantly in group I animals on day 0, 7, 15, or 21 (*p* > 0.05). In group II, the levels were highest on day 7, and there was no significant difference between sampling times (*p* > 0.05). BRSV antibody levels differed significantly between groups I and II on days 15 and 21 (*p* < 0.01 and *p* < 0.01, respectively). In group I, the levels decreased significantly in all animals before vaccination and then increased significantly in 11 animals (5/16) by day 15 after vaccination. By day 21, there was another decrease in all animals except for one calf. In group II, BRSV antibody levels showed decreases in 5 calves (5/16) on day 15 and in all 16 animals on day 21. Overall, the largest differences in antibody levels between groups I and II were observed for BRSV on day 15 and for all three agents on day 21 (Figure 2 and Table 7).

## 4. Discussion

To help newborn calves fight infectious diseases during their immunophysiological development, they must passively receive immune components such as immunoglobulins, immune cells, and certain molecules from their mothers through adequate amounts of colostrum within the first 24 h after birth. Calves that receive sufficient-quality colostrum during this time will generally have better health and growth during the pre-weaning phase compared to calves with inadequate colostrum intake [29,30,31,32]. In this study, changes in colostral antibody levels from birth to weaning were monitored. On day 2, BVDV, BoHV-1, and BRSV antibodies were detected in all calves that were seronegative on day 0, indicating that the transfer of colostral antibodies was successful. The highest level of colostral antibodies in the blood was detected on day 7, which indicates that antibody transfer to the blood continued after day 2. Results from another study showed that immunoglobulins can be absorbed in the neonatal intestinal tract even after 24 h after birth, the absorption rate continues even after 3 months, and the absorption rate is higher than the metabolic rate [32]. Similarly, a previous study reported that serum total IgG antibody titers increased in 7-day-old calves due to continued colostral antibody absorption [33].

In the present study, BoHV-1 antibody levels declined steadily until day 45 and then dropped to a critical level of 65% on day 55. Overall, BoHV-1 antibody levels decreased by 69.14% from day 7 to day 65. It has been reported that the colostrum of cattle naturally infected with BoHV-1 contains approximately three times more antibodies than blood samples and that newborn calves acquire passive immunity at a level similar to adult cattle due to colostrum intake from the first day of postnatal life [34]. The half-life of maternally transmitted BoHV-1 antibodies varies between 19 and 31.8 days [12]. Calves born to naturally infected mothers also have very low antibody titers at 3 months, although they reach seronegative status by 6 months [17]. Interestingly, our results show that antibody levels against all three viruses showed a decrease on day 35, with BVDV antibody levels falling the most, at 21%. It has been reported that calves switch from maternal antibody protection to endogenous IgG synthesis between 2 and 6 weeks of age, and the levels in their blood undergo an inversely proportional change [32,33]. Regarding overall monitoring, BVDV and BRSV antibody levels decreased less than those for BoHV-1 from day 7 to day 65, at 38% and 53%, respectively. Similarly, the authors of Ref. [33] reported that BVDV- and BRSV-specific maternal antibody concentrations decreased gradually from day 28 to day 91. Given that we did not detect BVDV, BoHV-1, or BRSV nucleic acid, it is likely that the decrease in antibody levels is not related to infection but to antibody catabolism, similar to a previous report [33]. During the monitoring period in our study, we observed several mild pneumonia cases (*M. haemolytica* and *P. multocida*) that responded to antibiotic treatment and subclinical BCoV in one calf. However, we cannot say that the intranasal vaccine administered at an early stage affected the serum BRSV antibody level, such as in the results described by Hill et al. [35]. Rather, based on our serological and virological data, it may have prevented an early infection with BRSV in the calves. Intranasal vaccines stimulate antiviral activity by inducing high interferon production in mucosal sites and serum and contribute to the development of the neonatal immune response [12]. BRSV nucleic acid has been detected at lower rates in vaccinated animals than in unvaccinated animals [36]. Furthermore, the combined administration of an intranasal virus vaccine enhanced with maternal antibody protection and vaccines containing subunits of two different respiratory tract bacteria may have contributed to the prevention of respiratory tract infection complications during the study period.

We observed that, in group I, the levels for all three virus-specific antibodies had decreased by day 7 just before the first vaccination. This decrease was sharper than on the monitoring days. We believe that this was caused by stress-induced cortisol levels, which may also have increased the rate of antibody catabolism. Future immunological studies can provide a better understanding of the relationship between stress and antibody catabolism.

In our study, antibody levels in group I were significantly lower than in group II on days 15 and 21, especially for BRSV-specific antibody levels on day 15. Whereas BoHV-1-specific antibody levels decreased in group I, they continued to show an increase in group II on day 21 after vaccination. Burns [37] hypothesized that stress is associated with poorer antibody responses to vaccination by affecting general processes such as antigen presentation, B lymphocyte clonal expansion, and the production of immunoglobulins. Stress-induced neuroendocrine changes suppress lymphocyte proliferation by increasing nitric oxide production from macrophages [38]. However, there is no evidence that it induces antibody catabolism, although Matty et al. [18] concluded that weaning stress may delay the antibody response provided by vaccines, thereby reducing its protective efficacy. In the present study, it is likely that the immunosuppressive effect of stress triggered by weaning and socialization is reflected in calf antibody levels. We found no evidence of active BoHV-1, BRSV, or BVDV infections during the sampling period, so we consider that stress had an adverse effect on antibody induction during vaccination in these calves.

Our findings show that antibody levels fell by day 21 after vaccination, which is the day of the second dose (days 21–28) recommended in some vaccine guidelines. However, these decreases were higher for both BoHV-1 and BRSV antibodies, especially in group I, which encountered earlier stress prior to vaccination. However, according to Ellis et al. [3], in order for a good primary immune response and a healthy anamnestic response to occur after the first vaccination, apoptosis of active cells playing a role in the primary response and a decrease in antibody titers are required; hence, there should be at least 3 weeks between the two doses. It is likely that the difference in antibody levels between the groups will be positively reflected in the antibody levels after the second (booster) vaccination in favor of group II. Although the interaction of vaccine strains with maternal antibodies should be taken into account [36], BoHV-1, BVDV-1, BVDV-2, and PIV-3 serum antibodies continue to decrease after the first dose of inactivated viral vaccine in calves with maternal antibodies, whereas higher antibodies against these viruses develop after the second dose of the vaccine [30,39]. On the other hand, studies have reported that high antibody levels against BRSV rapidly decrease after two doses of a modified live vaccine administered 21 days apart, although the reason for this is unknown [18,40]. Further studies on vaccine immunology will help us to better confirm this hypothesis.

## 5. Conclusions

Our data clearly demonstrate the critical role of maternal antibodies in providing protection against pathogens during the neonatal and weaning periods, when calves are most sensitive. In neonates, intranasal vaccines can provide precursor protection in the mucosal region, while systemic vaccination during the weaning period can provide holistic protection. However, while planning to maximize vaccine effectiveness, especially in calves, a vaccination strategy should be established by accounting for the presence of maternally derived antibodies. Hence, the first vaccination should be applied one week before socialization grouping to minimize the stress that may negatively affect vaccine titers. In addition, the first vaccination and booster vaccination interval should not be delayed during the weaning period, and the herd-based biosecurity approach should not be ignored. Otherwise, these infections will appear frequently, threatening animal health, herd productivity, and even the future of dairy cattle enterprises due to a domino effect.

## Figures and Tables

**Table 1:** Primers and probes used in RT-PCR for viruses.

Viruses	Primers and Probes (FAM/TAMRA) 5′-3′ Sequence	Reference
BoHV-1	F:TGTGGACCTAAACCTCACGGT R:GTAGTCGAGCAGACCCGTGTC P:FAM-AGGACCGCGAGTTCTTGCCGC-BHQ1	[21]
BVDV	Pesti3 F:CCTGAGTACAGGRTAGTCGTCA Pesti 4 R:GGCCTCTGCAGCACCCTATCA P:FAM-TGCYAYGTGGACGAGGGCATGC-BHQ-1	[22]
BRSV	F:AAGGGTCAAACATCTGCTTAACTAG R:TCTGCCTGWGGGAAAAAAG P:FAM-AGAGCCTGCATTRTCACAATACCACCCA- BHQ1	[23]
BCoV	F:CTGGAAGTTGGTGGAGTT R:ATTATCGGCCTAACATACATC P:FAM-CCTTCATATCTATACACATCAAGTTGTT-BHQ1	[24]
BPIV-3	F:TGTCTTCCACTAGATAGAGGGATAAAATT R:GCAATGATAACAATGCCATGGA P:FAM-ACAGCAATTGGATCAATAA-BHQ1	[25]

**Table 2:** Primers and probes used in conventional/RT-PCR for bacteria.

Bacteria	Target Genes	Gene Region 5′-3′ Sequence	Product Size (bp)	Reference
*M. haemolytica*	*sodA*	AGCAGCGACTACTCGTGTTGGTTCAG		[26]
	*sodA*	AAGACTAAAATCGGATAGCCTGAAACGCCTG		
	*sodA*	FAM-TTCAACCGCTAACCAGGACAACCCAC-BHQ1		
*P. multocida*	*16S rRNA*	CGCAGGCAATGAATTCTCTTC		
	*16S rRNA*	GGCGCTCTTCAGCTGTTTTT		
	*16S rRNA*	FAM-ACTGCACCAACAAATGCTTGCTGAGTTAGC-BHQ1		
*T. pyogenes*	*nanH*	CGCTAGTGCTGTAGCGTTGTTAAGT CCGAGGAGTTTTGACTGACTTTG	781	[27]
	*nanP*	TTGAGCGTACGCAGCTCTTC CCACGAAATCGGCCTTATTG	150	
	*cbpA*	GCAGGGTTGGTGAAAGAGTTTACT GCTTGATATAACCTTCAGAATTTGCA	124	
	*plo*	TCATCAACAATCCCACGAAGAG TTGCCTCCAGTTGACGCTTT	150	
*M. bovis*	*mb-mp1F*	TATTGGATCAACTGCTGGAT		[28]
	*mb-mp1R*	AGATGCTCCACTTATCTTAG	470	

**Table 3:** Relationships between colostrum and antibody titers on day 7.

		BVDV	BoHV-1	BRSV
Colostrum	r	0.046	−0.141	0.131
	*p*	NS	NS	NS

NS: non-significant; r: Pearson correlation coefficient; *p*: *p*-value.

**Table 4:** Percentage changes in antibody levels on each sampling day compared to day 7.

Antibody %	2nd	7th	15th	25th	35th	45th	55th	65th
BVDV	93.22	100.00	99.73	94.49	73.83	72.42	63.15	61.61
BoHV-1	78.97	100.00	90.25	82.29	73.74	62.27	34.89	30.86
BRSV	75.14	100.00	90.91	84.85	72.93	66.19	55.77	46.63

**Table 5:** Changes in BoHV-1, BVDV, and BRSV antibody levels (mean ± standard error *).

Days	BoHV-1 Antibody (S/P)	BVDV Antibody (S/P)	BRSV Antibody (V)
2nd	9.347 ± 0.495 ^de^	1.389 ± 0.024 ^b^	79.897 ± 1.748 ^c^
7th	7.381 ± 0.205 ^e^	1.490 ± 0.023 ^a^	106.334 ± 2.231 ^a^
15th	8.178 ± 0.268 ^de^	1.486 ± 0.024 ^a^	96.669 ± 2.048 ^b^
25th	8.969 ± 0.252 ^de^	1.408 ± 0.022 ^ab^	90.225 ± 1.790 ^b^
35th	10.009 ± 0.379 ^cd^	1.100 ± 0.019 ^c^	77.550 ± 1.364 ^cd^
45th	11.853 ± 0.431 ^c^	1.079 ± 0.015 ^c^	70.391 ± 1.182 ^d^
55th	21.153 ± 0.676 ^b^	0.941 ± 0.019 ^d^	59.300 ± 1.679 ^e^
65th	23.916 ± 0.738 ^a^	0.918 ± 0.016 ^d^	49.581 ± 2.165 ^f^
*p* < 0.001			

* a, b, c, d, e, f—Different lowercase superscripts indicate statistically significant difference between sampling days.

**Table 6:** Infectious agents detected from calves with respiratory disease.

Sample (S)	Socialization Groups						Sampling Days				Clinic
	Group I			Group II							
	7	15	21	7	15	21	35	45	55	65	
S.1			ND *				*P. multocida*				Pneumonia
S.2							*P. multocida*				Pneumonia
S.3			ND					*P. multocida*			Pneumonia
S.4								BCoV			Healthy
S.5			ND					*P. multocida*	*P. multocida*		Pneumonia
S.6								*P. multocida*	*P. multocida*		Pneumonia
S.7						ND			*M. haemolytica*	*M. haemolytica*	Pneumonia
S.8									*P. multocida*	*P. multocida*	Pneumonia
S.9	*M. haemolytica*										Pneumonia
S.10						ND					Pneumonia
S.11						ND					Pneumonia
S.12						ND					Pneumonia
S.13					ND						Pneumonia
S.14					ND						Pneumonia
S.15	ND										Pneumonia

* ND: Non determined.

**Table 7:** Antibody levels following socialization and vaccination.

Antibody	Group	Day 0	Day 7	Day 15	Day 21
BVDV	I	0.91 ± 0.03	0.71 ± 0.12	1.17 ± 0.09	0.98 ± 0.04
	II	0.92 ± 0.02	1.50 ± 0.04	1.67 ± 0.05	1.33 ± 0.05
BoHV-1	I	24.42 ± 1.12	42.31 ± 1.27	11.98 ± 0.67	26.17 ± 3.19
	II	23.41 ± 0.98	11.70 ± 0.39	9.04 ± 0.32	8.06 ± 0.28
BRSV	I	51.27 ± 2.90	28.93 ± 3.17	38.51 ± 2.57	29.24 ± 2.71
	II	47.89 ± 3.25	54.34 ± 3.00	51.86 ± 5.63	43.41 ± 2.32

## Data Availability

The data presented in this study are available in this article.

## Abbreviations

The following abbreviations are used in this manuscript.

BoHV-1	Bovine herpesvirus-1
BVDV	Bovine viral diarrhea virus
BPIV-3	Bovine parainfluenza virus-3
BRSV	Bovine respiratory syncytial virus
BCoV	Bovine coronavirus
ELISA	Enzyme-linked immunosorbent assay
*H. somni*	*Histophilus somni*
Ig	Immunoglobulin
*M. bovis*	*Mycoplasma bovis*
*M. haemolytica*	*Mannheimia haemolytica*
ND	Non determined
NS	Non-significant
*p*	*p*-value
*P. multocida*	*Pasteurella multocida*
r	Pearson correlation coefficient
RT-PCR	Real-time polymerase chain reaction
*T. pyogenes*	*Trueperella pyogenes*
